# A Phase 1 Study of mTORC1/2 Inhibitor BI 860585 as a Single Agent or with Exemestane or Paclitaxel in Patients with Advanced Solid Tumors

**DOI:** 10.3390/cancers12061425

**Published:** 2020-05-31

**Authors:** Filippo de Braud, Jean-Pascal H. Machiels, Daniela Boggiani, Sylvie W.H. Rottey, Matteo Duca, Marie Laruelle, Stefania Salvagni, Silvia Damian, Lore D.F. Lapeire, Marcello Tiseo, Alexandre Dermine, Mahmoud Ould-Kaci, Juergen Braunger, Juliane Rascher, Daniela Fischer, Josef Hoefler, Gabriella L. Mariani, Sara Cresta

**Affiliations:** 1 Fondazione IRCCS Istituto Nazionale dei Tumori, Medical Oncology Department, University of Milan, via G. Venezian, 1, 20133 Milan, Italy; 2 Institut Roi Albert II, Service d’Oncologie Médicale, Cliniques Universitaires Saint-Luc and Institut de Recherche Clinique et Expérimentale (Pole MIRO), Université Catholique de Louvain, Avenue Hippocrate 10, 200 Woluwe-Saint-Lambert, 1200 Brussels, Belgium; jean-pascal.machiels@uclouvain.be; 3 Medical Oncology Unit, University Hospital of Parma, Via Antonio Gramsci 14, 43126 Parma, Italy; boggiani@ao.pr.it (D.B.); mtiseo@ao.pr.it (M.T.); 4 Drug Research Unit Ghent, Ghent University Hospital, Corneel Heymanslaan 10, 9000 Ghent, Belgium; sylvie.rottey@ugent.be (S.W.H.R.); lore.lapeire@uzgent.be (L.D.F.L.); 5 Medical Oncology Department, Fondazione IRCCS Istituto Nazionale dei Tumori, via G. Venezian, 1, 20133 Milan, Italy; matteo.duca@istitutotumori.mi.it (M.D.); silvia.damian@istitutotumori.mi.it (S.D.); sara.cresta@istitutotumori.mi.it (S.C.); 6 Institut Roi Albert II, Cliniques Universitaires Saint-Luc and Institut de Recherche Clinique et Expérimentale (Pole MIRO), Université catholique de Louvain, Avenue Hippocrate 10, 200 Woluwe-Saint-Lambert, 1200 Brussels, Belgium; laruelle.marie@gmail.com (M.L.); alexandre.dermine@jolimont.be (A.D.); 7 Policlinico S. Orsola Malphigi, via Giuseppe Massarenti, 13, 40138 Bologna, Italy; stefania.salvagni@aosp.bo.it; 8 Boehringer Ingelheim Pharmaceuticals, Inc., 900 Ridgebury Rd, Ridgefield, CT 06877, USA; mahmoud.ould_kaci@boehringer-ingelheim.com; 9 Boehringer Ingelheim Pharma GmbH & Co. KG, Birkendorfer Str. 65, 88397 Biberach an der Riß, Germany; juergen.braunger@boehringer-ingelheim.com (J.B.); juliane.rascher.ext@boehringer-ingelheim.com (J.R.); daniela.fischer@boehringer-ingelheim.com (D.F.); 10 Staburo GmbH, Aschauer Str. 26b, 81549 Munich, Germany, on behalf of Boehringer Ingelheim Pharma GmbH & Co. KG, Biberach, Germany; josef.hoefler.ext@boehringer-ingelheim.com; 11 Boehringer Ingelheim, via Giovanni Lorenzini, 8, 20139 Milan, Italy; gabriella.mariani@astrazeneca.com

**Keywords:** mTOR serine-threonine kinases, BI 860585, clinical trial, phase 1, maximum tolerated dose, pharmacokinetics

## Abstract

This phase 1 trial (NCT01938846) determined the maximum tolerated dose (MTD) of the mTOR serine/threonine kinase inhibitor, BI 860585, as monotherapy and with exemestane or paclitaxel in patients with advanced solid tumors. This 3+3 dose-escalation study assessed BI 860585 monotherapy (5–300 mg/day; Arm A), BI 860585 (40–220 mg/day; Arm B) with 25 mg/day exemestane, and BI 860585 (80–220 mg/day; Arm C) with 60–80 mg/m^2^/week paclitaxel, in 28-day cycles. Primary endpoints were the number of patients with dose-limiting toxicities (DLTs) in cycle 1 and the MTD. Forty-one, 25, and 24 patients were treated (Arms A, B, and C). DLTs were observed in four (rash (*n* = 2), elevated alanine aminotransferase/aspartate aminotransferase, diarrhea), four (rash (*n* = 3), stomatitis, and increased gamma-glutamyl transferase), and two (diarrhea, increased blood creatine phosphokinase) patients in cycle 1. The BI 860585 MTD was 220 mg/day (Arm A) and 160 mg/day (Arms B and C). Nine patients achieved an objective response (Arm B: Four partial responses (PRs); Arm C: Four PRs; one complete response). The disease control rate was 20%, 28%, and 58% (Arms A, B, and C). The most frequent treatment-related adverse events (AEs) were hyperglycemia (54%) and diarrhea (39%) (Arm A); diarrhea (40%) and stomatitis (40%) (Arm B); fatigue (58%) and diarrhea (58%) (Arm C). The MTD was determined in all arms. Antitumor activity was observed with BI 860585 monotherapy and in combination with exemestane or paclitaxel.

## 1. Introduction

The PI3K/AKT/mTOR pathway plays an important role in the regulation of metabolism, survival, and proliferation of mammalian cells [1,2,3]. This pathway is often hyperactivated in human cancers and has been associated with resistance to conventional therapy [4].

mTOR comprises at least two different complexes, the mTOR complex 1 (rapamycin-sensitive mTORC1) and complex 2 (mTORC2) [5]. As the main downstream effectors in the PI3K/AKT pathway, the mTOR complexes are central factors in the regulation of cell growth, proliferation, metabolism, angiogenesis, and cell survival processes [3]. Thus, targeting the mTOR complexes could play an important role in cancer therapy [6].

First-generation mTOR inhibitors (rapalogs), derived from the immunosuppressive drug rapamycin, demonstrated limited therapeutic success [7], exhibiting only partial inhibition of mTORC1 signaling, and no inhibition of mTORC2 or feedback-activation of PI3K/AKT signaling via S6K and mTORC2 activity [8]. A new generation of ATP-competitive mTOR inhibitors currently in clinical development were designed to target the kinase domains of mTOR and fully inhibit both mTORC1 and mTORC2 [7]. BI 860585 is a potent, selective, ATP-competitive mTORC1 and mTORC2 serine/threonine kinase inhibitor showing strong preclinical efficacy against various sarcoma types [9].

Although data from early clinical trials with rapalogs and other mTOR inhibitors have demonstrated modest response rates as single agents [10,11], mTOR inhibition in combination with chemotherapy, hormone therapy, and other targeted therapies may be more effective than monotherapy. These combinations are hypothesized to induce tumor regression and could circumvent acquired resistance. This hypothesis is supported by preclinical and clinical studies with different rapalogs (e.g., temsirolimus and everolimus) in advanced cancers [4,12,13]. Of note, in the phase 3 BOLERO-2 study, everolimus in combination with the aromatase inactivator, exemestane, significantly improved PFS versus exemestane alone in postmenopausal women with hormone-receptor (HR)-positive advanced breast cancer who had relapsed or progressed on nonsteroidal aromatase inhibitors [4]. This treatment combination has since been approved by the US Food and Drug Administration (FDA) and European Medicines Agency (EMA) for use in this setting.

To investigate the potential for combining mTOR-complex inhibition with current standard therapies, this phase 1, dose finding, first-in-human study evaluated BI 860585 as monotherapy and in combination with either exemestane or paclitaxel in heavily pretreated patients with advanced and/or metastatic solid tumors. Maximum tolerated doses (MTDs) of BI 860585 as monotherapy, and combined with exemestane or paclitaxel, were identified; adverse events (AEs) were generally predictable and manageable. Further, antitumor activity was seen in all treatment arms.

## 2. Results

### 2.1. Patients and Treatment Exposure

The first patient was enrolled on 10 September, 2013 and the last patient was enrolled on 02 June, 2016. A total of 41 patients were treated in Arm A, 25 in Arm B, and 24 in Arm C. Patient baseline characteristics are shown in Table 1. The median age was 61 years (range 20–79 years) and most patients (95.6%) had an Eastern Cooperative Oncology Group performance status (ECOG PS) < 2. The majority of patients for whom data were available had ≥3 metastatic sites at screening and had received at least one prior line of either chemotherapy, radiotherapy, and/or prior surgery.

Patients received BI 860585 5–300 mg/day as a single agent (Arm A), 40–220 mg/day in combination with exemestane (Arm B), and 80–220 mg/day in combination with paclitaxel (Arm C). At the time of data analysis (27 November, 2017), all patients had discontinued trial medication. Reasons for discontinuation included progressive disease (66.7%), AEs (16.7%), and patient withdrawal (5.6%). The median (range) treatment exposure to BI 860585 was 56 (17–561) days in Arm A, 56 (7–784) days in Arm B, and 125 (13–448) days in Arm C. The median (range) treatment exposure to exemestane (Arm B), and paclitaxel (Arm C) was 63 (14–791) days and 14.5 (3–32) infusions, respectively.

### 2.2. MTD Determination

Four evaluable patients treated in Arm A experienced a dose-limiting toxicity (DLT) during cycle 1 (all grade 3; Table 2); rash (120 mg (*n* = 1)), elevated alanine aminotransferase/aspartate aminotransferase (160 mg (*n* = 1)), diarrhea, and rash (both 300 mg (*n* = 2)). The MTD of BI 860585 was 220 mg/day (Table 2).

Four treated and evaluable patients in Arm B experienced DLTs; grade 3 rash (120 mg (*n* = 1) and 220 mg (*n* = 2)), and grade 3 stomatitis and increased gamma-glutamyl transferase (160 mg; *n* = 1). The MTD of BI 860585 in combination with exemestane was 160 mg/day.

Two evaluable patients treated in Arm C experienced a DLT during cycle 1 (both grade 3); diarrhea and increased blood creatine phosphokinase (both at 220 mg). The MTD of BI 860585 in combination with paclitaxel was 160 mg/day.

### 2.3. Safety

All patients experienced at least one AE, including laboratory abnormalities, and most patients experienced a treatment-related AE (88% in Arm A, 96% in Arm B, and 100% in Arm C (Table 3)). The most frequent treatment-related AEs were hyperglycemia (54%; *n =* 22), diarrhea (39%; *n =* 16), and nausea (37%; *n =* 15) in Arm A; diarrhea (40%; *n =* 10), stomatitis (40%; *n =* 10), hyperglycemia (36%; *n =* 9), and fatigue (36%; *n =* 9) in Arm B; fatigue (58%; *n =* 14), diarrhea (58%; *n =* 14), hyperglycemia (54%; *n =* 13), anemia (50%; *n =* 12), and decreased appetite (46%; *n =* 11) in Arm C. The majority of treatment-related AEs were of grade ≤3 in severity; there were no grade 5 treatment-related AEs. One patient experienced a transient grade 3 elevation of blood creatine phosphokinase (DLT), but there were no clinical manifestations suggestive of adverse musculoskeletal effects in this patient.

A total of 22 patients (54%) in Arm A, 10 patients (40%) in arm B, and 13 patients (54%) in Arm C had serious AEs. Seven (17%) patients in Arm A, nine (36%) patients in Arm B, and six (25%) patients in Arm C experienced AEs leading to dose reduction of BI 860585. Thirteen (32%) patients in Arm A, seven (28%) patients in Arm B, and five (21%) patients in Arm C experienced AEs leading to discontinuation of BI 860585.

### 2.4. Antitumor Activity

Best overall tumor responses for each treatment arm are shown in Table 4 and Supplementary Figure S1, including the percentage change from baseline in target lesions. The objective response rate (ORR) was 0%, 16% (four partial responses (PRs) including an estrogen-receptor-positive breast cancer patient who received a first-line combination of ridaforolimus, dalotuzumab, and exemestane) and 21% (one complete response (CR) and four PRs) in Arms A, B, and C, respectively (Table 4). The patient achieving a CR was a 69-year-old female with breast cancer who had received approximately 10 prior lines of therapy including: Epirubicin, CMF (5-FU, methotrexate, and capecitabine) carboplatin and paclitaxel; capecitabine and vinorelbine, nabpaclitaxel, exemestane plus everolimus; and multiple lines of hormone therapies. At screening for this trial, she presented with skin metastases and soft tissue involvement. At baseline; this patient did not have measurable disease/target lesions and only one nontarget lesion was documented. The patient received BI 860585 220 mg plus 80 mg paclitaxel for six cycles and achieved a CR, measured in the nontarget lesion, on day 120, which was maintained for 63 days. The median (range) duration of objective response was eight months (0–23) for Arm B and five months (2–9) for Arm C. One and three of the responding patients in Arm B and Arm C, respectively, had received ≥3 lines of previous chemotherapy. The disease control rate (DCR; CR + PR + stable disease (SD)) was 20% in Arm A, 28% in Arm B, and 58% in Arm C. The median (range) duration of disease control was eleven (4–17), nine (2–25), and seven (4–15) months for Arms A, B, and C, respectively (Table 4).

### 2.5. Pharmacokinetics

Peak plasma concentrations (C_max_) occurred 2–6 h following once-daily oral administration of BI 860585 (5–300 mg) in Arm A. The trough BI 860585 plasma concentration did not increase after day 8, indicating that a steady state had been reached (Figure S2). Repeated daily dosing resulted in 1.89-fold accumulation of BI 860585 area under the curve (AUC) values at steady state. Inter-individual variability of the AUC at steady state at the MTD in Arm A was moderate (geometric coefficient of variation 24.3%). BI 860585 plasma exposure was almost dose-proportional following a single-dose administration across the complete dose range tested (5–300 mg), and after multiple dosing between 40 and 220 mg (Table S1; Figure S2). See Table S2 for the pharmacokinetic characteristics at the MTD of the three arms. 

Pharmacokinetics were determined in preselected patients enrolled in Arm A (120, 160, and 220 mg dose cohorts) with and without food (see Table S3 for proposed composition of standard continental breakfast) on day 1 and day 2 of the first treatment cycle. The presence of food had an influence on the rate of absorption, i.e., caused a delay and a reduction of less than 20% in C_max_. The effect of food on the extent of absorption was minimal, with the AUC_0–24_ being reduced by less than 10%.

In Arm A, dose proportionality was confirmed after single administration. Exposure at steady state increased with the dose in an almost proportional manner over the dose range tested (5–300 mg). In Arms B and C, dose proportionality at steady state could be established for the dose ranges tested (40–220 mg BI 860585 in Arm B, and 80–220 mg BI 860585 in Arm C). 

### 2.6. Biomarker Assessment

In all three treatment arms, median reductions in AKT phosphorylation at Ser473 (pAKT)/AKT ratios of 45–54% were observed in platelet-rich plasma blood samples within 3 h following BI 860585 doses of 120 mg or greater (Figure S3). At steady state, the inhibition persisted for all three treatment arms. There was no clear indication of a pharmacokinetic/pharmacodynamic correlation, or a correlation between pAKT/AKT ratio reductions and disease control. Data for BI 860585 in combination with exemestane and paclitaxel were consistent with monotherapy.

## 3. Discussion

This phase 1 study demonstrated that the mTOR inhibitor, BI 860585, is tolerable as monotherapy or combined with standard treatments. The MTDs established in this study were BI 860585 220 mg/day as monotherapy, BI 860585 160 mg/day plus exemestane 25 mg/day, and BI 860585 160 mg/day plus paclitaxel 80 mg/m^2^/week. Safety was similar across the three treatment arms, with AEs that were generally manageable and consistent with the mechanisms of action of the study drugs. The most common DLTs were rash and diarrhea (Table 2), and the most frequently reported treatment-related AEs included hyperglycemia, diarrhea, nausea, rash, fatigue, and stomatitis (Table 3). These are consistent with previous reports of mTOR-inhibitor treatment of advanced solid tumors [14,15]. Higher incidences of anemia and neutropenia were observed in patients receiving BI 860585 combined with paclitaxel compared with those receiving BI 860585 alone or in combination with exemestane. These AEs are consistent with the known safety profile of paclitaxel [16].

Signs of antitumor activity across various tumor types were observed with BI 860585 monotherapy and with the combination therapies (Table 4; Figure S1). Objective responses were observed with BI 860585 in combination with exemestane (16%) or paclitaxel (21%); in the monotherapy arm, 20% of patients had SD. These results are particularly encouraging considering that most patients who responded had been heavily pretreated with chemotherapy or hormonal cancer treatment. Given the small patient numbers, it is challenging to suggest a patient population that could derive a particular benefit from BI 860585 in combination with exemestane or paclitaxel; however, responses were seen in two patients with breast cancer, a patient population for which the combination of the mTOR inhibitor everolimus is approved in combination with exemestane. It is also encouraging that one of the patients responding had progressed on prior mTORC1-based therapy, suggesting that BI 860585 was able to resensitize the tumor through dual mTORC1/2 inhibition; however, this would need to be confirmed in larger studies.

Pharmacokinetic analyses demonstrated almost dose-proportional plasma exposure during BI 860585 monotherapy following single administration and after multiple dosing. The rate of absorption of BI 860585 was rapid, and although the presence of food had a slight effect on the rate of absorption, the extent of absorption was similar in the fasting or fed state (Table S1). A reduction in C_max_ after food has been described for other mTOR targeted agents [17]. Taken together, the safety and pharmacokinetic data suggest that treatment with BI 860585 as monotherapy or in combination with exemestane or paclitaxel is feasible and tolerable for patients with advanced solid tumors.

Biomarker analyses were conducted to evaluate the biological activity of BI 860585. Decreased AKT phosphorylation at Ser473 has been identified as a pharmacodynamic biomarker for mTORC2 inhibition [18,19]. In this study, a 45%–54% reduction in pAKT/AKT ratios was observed in platelet-rich plasma blood samples within 3 h of dosing in all treatment arms (Figure S3), suggesting that treatment with BI 860585 has an impact on the intended molecular target. The reduction of pAKT persisted at steady-state concentrations of BI 860585. There was no clear correlation between pharmacokinetics/pharmacodynamics or between reductions in pAKT/AKT ratios and disease control, likely due to the high level of variability in the data, together with the small sample sizes. Biomarker findings with BI 860585 in combination with exemestane and paclitaxel were consistent with monotherapy.

A dose-expansion stage was originally planned, but the development of BI 860585 was discontinued during this study due to a strategic decision made by the sponsor, and consequently, the dose-expansion stage was cancelled. Nevertheless, the results of this trial will contribute to informed decision-making about the clinical development of other mTOR inhibitors. Given the mode of action and available clinical data for compounds targeting the PI3K/AKT/mTOR pathway, including the preliminary antitumor activity shown in this trial, it is expected that a combination strategy either with endocrine, chemotherapy, and/or other targeted therapies may result in the most efficacious use of this class of drug. Further studies of mTOR inhibitors are warranted to evaluate these regimens in relevant patient populations, particularly if a predictive marker can be defined to select patients who would gain the most from this treatment approach. 

## 4. Methods

### 4.1. Patients

Eligible patients were aged ≥18 years and had advanced nonresectable and/or metastatic solid tumors, disease progression, and an ECOG PS ≤ 2. Full patient eligibility criteria for all treatment arms are included in the Supplementary Methods and Table S4.

The study was conducted in accordance with the Declaration of Helsinki and Good Clinical Practice guidelines and the study protocol (study protocol code 1325.1) was approved by the Institutional Review Boards of the participating institutions. Written informed consent was obtained from all patients.

### 4.2. Study Design and Treatment

**Figure 1:**
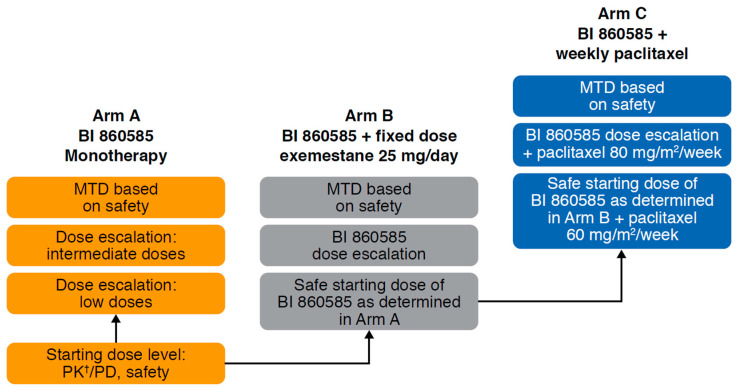
Study design. Abbreviations: MTD: Maximum tolerated dose; PD: Pharmacodynamics; PK: Pharmacokinetics. ^†^ Including food effects.

This multicenter, open-label, phase 1 trial (NCT01938846) employed a 3+3 dose escalation design to determine the MTD, safety, pharmacokinetics, and antitumor activity of BI 860585 as monotherapy and in combination with exemestane or paclitaxel in patients with advanced and/or metastatic solid tumors. The study was conducted at four study sites in Italy and Belgium and recruitment was staggered across three distinct treatment arms running in parallel (Figure 1). In the monotherapy arm (Arm A), patients received oral continuous daily dosing of BI 860585 at a starting dose of 5 mg/day over a 28-day cycle. 

Enrolment in the combination Arm B opened when the first treatment-related AE of grade ≥2 had been observed in Arm A, and the starting dose was determined by the Safety Monitoring Committee (SMC) as 40 mg, i.e., the dose level at which no drug-related grade ≥2 AE had been observed in Arm A. Enrolment in the combination Arm C opened when the SMC had determined a safe starting dose of 80 mg, i.e., the dose level at which no drug-related grade ≥3 AE had been observed in the dose escalation of Arm B. Patients were assigned to the individual combination arms (Arm B and Arm C) based on the investigators’ clinical judgment. In Arm B, patients received oral exemestane 25 mg/day (standard fixed dose) continuously on a 28-day cycle. In Arm C, patients received an intravenous infusion of paclitaxel 60 mg/m^2^ in the first dose level cohort, then escalated to 80 mg/m^2^ (standard dose) once weekly on a 28-day cycle. Treatment with exemestane or paclitaxel began on day 7 of cycle 1. Patients were eligible for repeated cycles until disease progression or intolerable AEs.

The primary endpoints for this study were the MTD and the number of DLTs in each treatment arm. The MTD was based on the number of patients with a DLT during the first cycle, and was defined as the dose at which no more than one of six patients experienced a DLT during cycle 1 or the dose level below which ≥2 of six patients experienced a DLT during cycle 1. A DLT was defined as any of the following: Grade 4 neutropenia lasting ≥7 days; grade ≥3 febrile neutropenia/neutropenia with documented infection; grade 3 thrombocytopenia associated with bleeding requiring transfusion; grade 4 thrombocytopenia or anemia; any grade ≥3 nonhematologic toxicity that persisted despite adequate medical intervention or prophylaxis; any grade 3 hyperglycemia that did not recover to grade ≤1 within two weeks of adequate therapy; and any toxicity resulting in a >14 day delay in starting cycle 2.

Other endpoints included ORR and DCR, both per Response Evaluation Criteria in Solid Tumors (RECIST) criteria version 1.1 [20], duration of objective response/clinical benefit, safety, and pharmacokinetic parameters of BI 860585, administered as a single agent and in combination regimens, with or without food, at the MTD for each arm. 

Detailed definitions and methodology for secondary assessments and pharmacokinetic parameters are described in the Supplementary Methods.

### 4.3. Safety Analyses

Safety was assessed by monitoring AEs (National Cancer Institute Common Terminology Criteria for Adverse Events version 4.03), clinical laboratory parameters, electrocardiograms, and vital signs. Patients were included in the safety analysis if they had taken at least one dose of any trial medication. Patients were evaluable for DLTs if they had been observed for at least the first treatment cycle and had undergone all of the safety assessments.

### 4.4. Pharmacokinetics

Plasma concentration-time profiles and pharmacokinetic parameters of BI 860585 were determined in all patients who received oral doses of 5–220 mg. In addition, an exploratory analysis of the effect of food was conducted for the BI 860585 monotherapy arm. The following definition of a standard continental breakfast was given in order to standardize the food intake prior to assessing food effects on BI 860585: One egg, two bread rolls, 20 g butter, 25 g cheese, 25 g ham/saveloy, 25 g jam, and one cup (~250 mL) of decaffeinated tea or coffee (average energy value per breakfast: 688 kcal or 2880 kJ). Alternative food components/quantity could be proposed by the investigator but the caloric breakdown of the test meal had to be consistent with the one indicated. See Supplementary Table S3 for the proposed composition of the standard continental breakfast. Pharmacokinetic parameters of interest were half-life (T_1/2_), time to reach maximum plasma concentration (T_max_), C_max_, and AUC for BI 860585 after single dosing and at steady state, when administered as a single agent and in combination with exemestane or paclitaxel.

### 4.5. Biomarker Analyses

Decreased pAKT is a pharmacodynamic biomarker for mTORC2 inhibition [18,19]. As such, exploratory analyses of pAKT were conducted using platelet-rich plasma blood samples from patients in the different treatment arms (Supplementary Methods; Table S5).

### 4.6. Statistical Analysis

All patients who were treated with at least one dose of BI 860585 were included in the analyses of safety and efficacy. All statistical analyses were descriptive and exploratory, and no formal statistical tests were performed.

## 5. Conclusions

The current phase 1 study identified the MTDs of BI 860585 as monotherapy (220 mg/day), and combined with exemestane (BI 860585 160 mg/day plus exemestane 25 mg/day) or paclitaxel (BI 860585 160 mg/day plus paclitaxel 80 mg/m^2^/week). These treatments had manageable safety profiles, and DLTs and treatment-related AEs were consistent with previous reports of mTOR-inhibitor treatment of advanced solid tumors [14,15]. Further, both monotherapy and combination regimens showed evidence of antitumor activity in this heavily pretreated patient population, with objective responses observed in the combination arms.

## Figures and Tables

**Table 1:** Baseline characteristics.

Characteristic	Arm A	Arm B	Arm C
	(*n =* 41)	(*n =* 25)	(*n =* 24)
	BI 860585	BI 860585 + Exemestane	BI 860585 + Paclitaxel
Gender, *n* (%)			
Male	28 (68)	2 (8)	12 (50)
Female	13 (32)	23 (92)	12 (50)
Median age, years (range)	61 (20–79)	61 (40–77)	61 (36–76)
ECOG PS, *n* (%)			
0	19 (46)	14 (56)	11 (46)
1	20 (49)	10 (40)	12 (50)
2	2 (5)	1 (4)	1 (4)
Advanced solid tumor classification^†^, *n* (%)			
Breast	2 (5)	9 (36)	4 (17)
Colorectal	8 (20)	0	0
Head and neck	6 (15)	3 (12)	2 (8)
Kidney	4 (10)	0	4 (17)
Liver	3 (7)	0	0
Ovary	1 (2)	5 (20)	3 (13)
Other ^‡^	17 (41)	8 (32)	11 (46)
Number of metastatic sites at screening, *n* (%)			
<3	20 (49)	9 (36)	11 (46)
≥3	21 (51)	15 (60)	10 (42)
Unknown	0	1 (4)	3 (13)
Number of patients with prior chemotherapy, *n* (%)	40 (98)	21 (84)	19 (79)
<3 prior chemotherapy lines	17 (42)	6 (24)	8 (33)
≥3 prior chemotherapy lines	23 (56)	15 (60)	11 (46)
Unknown	1 (2)	4 (16)	5 (21)
Prior radiotherapy, *n* (%)			
No	19 (46)	6 (24)	13 (54)
Yes	22 (54)	19 (76)	11 (46)

Abbreviations: ECOG PS: Eastern Cooperative Oncology Group performance status. ^†^ Tumor type specified if *n* ≥ 3 patients. ^‡^ Other tumor classifications were, in Arm A: Anal region (1), biliary tree (2), bladder (2), gastrointestinal tract (1), lung (2), non-small cell lung cancer (1), not specified (2), pancreas (2), prostate (1), small intestine (1), stomach (1), testis (1); in Arm B: Cervix (1), carcinoma of unknown primary site (1), endometrial cancer (2), genitourinary system (1), gynecologic (1), ureter (1), uterine malignancy (1); in Arm C: Adrenal (1), bladder (1), cervix (2), carcinoma of unknown primary site (1), gynecologic (1), lung (1), pancreas (2), prostate (1), stomach (1).

**Table 2:** Dose-limiting toxicity (DLT) in cycle 1.

Arm A: BI 860585 Monotherapy			Arm B: BI 860585 + Exemestane (25 mg/day)		Arm C: BI 860585 + Paclitaxel (60–80 mg/m^2^/week)	
BI 860585 Dose, mg	N (MTD Evaluable)	MTD-Evaluable Patients with DLTs*, *n*	N (MTD Evaluable)	MTD-Evaluable Patients with DLTs*, *n*	N (MTD Evaluable)	MTD-Evaluable Patients with DLTs^†^, *n*
5	3 (3)	0	–	–	–	–
10	3 (3)	0	–	–	–	–
20	3 (3)	0	–	–	–	–
40	3 (3)	0	3 (3)	0	–	–
80	3 (3)	0	4 (3)	0	7 (6)	0
120	7 (6)	1 (rash)	7 (6)	1 (rash)	7 (3)	0
160	7 (6)	1 (elevated AST/ALT)	8 (6)	1 (stomatitis and increased GGT)	7 (6)	0
220	9 (6)	0	3 (3)	2 (rash)	3 (3)	1 (diarrhea) 1 (increased blood CP)
300	3 (3)	2 (diarrhea (*n =* 1); rash (*n =* 1))	–	–	–	–

Abbreviations: DLT: Dose-limiting toxicity; ALT: Alanine aminotransferase; AST: Aspartate aminotransferase; CP: Creatine phosphokinase; GGT: Gamma-glutamyl transferase; MTD: Maximum tolerated dose. ^†^ All DLTs among MTD-evaluable patients were grade 3.

**Table 3:** Treatment-related adverse events (AEs) occurring in ≥10% of patients in any one treatment arm.

AE, *n* (%)	Arm A (*n =* 41)		Arm B (*n =* 25)		Arm C (*n =* 24)	
	BI 860585		BI 860585 + Exemestane		BI 860585 + Paclitaxel	
	All Grade	Grade ≥ 3	All Grade	Grade ≥ 3	All Grade	Grade ≥ 3
Any treatment-related AEs	36 (88)	11 (27)	24 (96)	9 (36)	24 (100)	12 (50)
Hyperglycemia	22 (54)	1 (2)	9 (36)	1 (4)	13 (54)	0
Diarrhea	16 (39)	2 (5)	10 (40)	2 (8)	14 (58)	4 (17)
Nausea	15 (37)	0	5 (20)	0	9 (37)	0
Rash	11 (27)	3 (7)	8 (32)	4 (16)	7 (29)	1 (4)
Fatigue	10 (24)	0	9 (36)	2 (8)	14 (58)	2 (8)
Decreased appetite	9 (22)	0	7 (28)	0	11 (46)	0
Stomatitis	9 (22)	0	10 (40)	2 (8)	7 (29)	0
Vomiting	9 (22)	0	3 (12)	0	4 (17)	0
Hypertriglyceridemia	8 (20)	0	0	0	2 (8)	0
Hypercholesterolemia	6 (15)	0	4 (16)	0	6 (25)	0
Creatine phosphokinase increased	6 (15)	0	6 (24)	0	3 (13)	1 (4)
Asthenia	4 (10)	0	3 (12)	0	3 (13)	0
Lipase increased	4 (10)	1 (2)	3 (12)	0	1 (4)	0
Pruritus	3 (7)	1 (2)	5 (20)	0	5 (21)	0
AST increased	3 (7)	2 (5) ^†^	4 (16)	0	2 (8)	0
ALT increased	2 (5)	2 (5) ^†^	3 (12)	0	2 (8)	0
Polyneuropathy	2 (5)	0	0	0	7 (30)	2 (8)
Anemia	1 (2)	0	1 (4)	0	12 (50)	2 (8)
Dry mouth	1 (2)	0	4 (16)	0	3 (13)	0
Dry skin	1 (2)	0	0	0	5 (21)	0
Abdominal pain	1 (2)	0	1 (4)	1 (4)	4 (17)	0
Weight decreased	1 (2)	0	1 (4)	0	5 (21)	0
Hypomagnesemia	1 (2)	0	0	0	4 (17)	1 (4)
Neutropenia	0	0	0	0	7 (29)	1 (4) ^†^
White blood cell count decreased	0	0	1 (4)	0	6 (25)	4 (17)
Alopecia	0	0	0	0	5 (21)	0
Paresthesia	1 (2)	0	0	0	3 (13)	0
Epistaxis	0	0	0	0	4 (17)	0
Paronychia	0	0	0	0	3 (13)	0

Abbreviations: AEs: Adverse events; ALT: Alanine aminotransferase; AST: Aspartate aminotransferase. ^†^ One grade 4 event.

**Table 4:** Best overall tumor response during treatment.

Best Overall Tumor Response	Arm A (*n =* 41)	Arm B (*n =* 25)	Arm C (*n =* 24)
	BI 860585	BI 860585 + Exemestane	BI 860585 + Paclitaxel
Disease control/clinical benefit, *n* (%)	8 (20)	7 (28)	14 (58)
Complete response	0	0	1 (4)
Partial response	0	4 (16) ^†^	4 (17)
Stable disease	8 (20)	3 (12)	9 (38)
Objective response (complete + partial)	0	4 (16) ^‡^	5 (21) ^§^
Median duration of disease control, months (range)	11 (4–17)	9 (2–25)	7 (4–15)
Median duration of objective response, months (range)	–	8 (0–23)	5 (2–9)
Progressive disease, *n* (%)	23 (56)	12 (48)	6 (25)
Not evaluable, *n* (%)	10 (24)	6 (24)	4 (17)

^†^ Including an estrogen-receptor-positive breast cancer patient pretreated with a first-line combination of ridaforolimus (mTORC1 inhibitor) + dalotuzumab (anti-IGF1R (insulin-like growth factor 1 receptor)) + exemestane. ^‡^ Three patients had <3 lines of prior chemotherapy and one patient had ≥3 lines of prior chemotherapy. ^§^ Two patients had <3 lines of prior chemotherapy and three patients had ≥3 lines of prior chemotherapy.

## Supplementary Materials

The following are available online at https://www.mdpi.com/2072-6694/12/6/1425/s1, Figure S1: (a) Best percentage change from baseline in target lesions (Arm A: Monotherapy); (b) best percentage change from baseline in target lesions (Arm B: Combination with exemestane); (c) best percentage change from baseline in target lesions (Arm C: Combination with paclitaxel); Figure S2: Plasma concentration-time profiles after once-daily administration of 5 to 300 mg BI 860585 (semi-logarithmic scale); Table S1: Statistical evaluation of the effect of food on exposure to BI 860585 monotherapy; Table S2: Steady-state pharmacokinetic characteristics obtained at day 22 at the MTD in all arms; Table S3: Proposed composition of the standard continental breakfast; Table S4: Key inclusion and exclusion criteria; and Table S5: Blood sampling schedule for pharmacokinetic analyses during cycle 1.

## Data sharing statement

To ensure independent interpretation of clinical study results, Boehringer Ingelheim grants all external authors access to all relevant material, including participant-level clinical study data, and relevant material as needed by them to fulfill their role and obligations as authors under the ICMJE criteria. Furthermore, clinical study documents (e.g., study report, study protocol, statistical analysis plan) and participant clinical study data are available to be shared after publication of the primary manuscript in a peer-reviewed journal and if regulatory activities are complete and other criteria met per the BI Policy on Transparency and Publication of Clinical Study Data: https://trials.boehringer-ingelheim.com/transparency_policy.html. Prior to providing access, documents will be examined, and, if necessary, redacted and the data will be de-identified, to protect the personal data of study participants and personnel, and to respect the boundaries of the informed consent of the study participants. Clinical Study Reports and Related Clinical Documents can be requested via this link: https://trials.boehringer-ingelheim.com/trial_results/clinical_submission_documents.html. All such requests will be governed by a Document Sharing Agreement. Bona fide, qualified scientific and medical researchers may request access to de-identified, analyzable participant clinical study data with corresponding documentation describing the structure and content of the datasets. Upon approval, and governed by a Data Sharing Agreement, data are shared in a secured data-access system for a limited period of one year, which may be extended upon request. Researchers should use https://trials.boehringer-ingelheim.com to request access to study data.
