# Supplementary material for: A Phase 1 Study of mTORC1/2 Inhibitor BI 860585 as a Single Agent or with Exemestane or Paclitaxel in Patients with Advanced Solid Tumors
Source: Cancers (Basel). 2020 May 31;12(6):1425. doi: 10.3390/cancers12061425 (PMC7352719; doi:10.3390/cancers12061425)
Supplement: Supplementary file 1 [file cancers-12-01425-s001.pdf]

Supplementary Materials: A Phase 1 Study of mTORC1/2 Inhibitor BI 860585 as a Single Agent or with Exemestane or Paclitaxel in Patients with Advanced Solid Tumors

Filippo de Braud, Jean-Pascal H. Machiels, Daniela Boggiani, Sylvie W.H. Rottey, Matteo Duca, Marie Laruelle, Stefania Salvagni, Silvia Damian, Lore D.F. Lapeire, Marcello Tiseo, Alexandre Dermine, Mahmoud Ould-Kaci, Juergen Braunger, Juliane Rascher, Daniela Fischer, Josef Hoefler, Gabriella L. Mariani and Sara Cresta

Patients

(1) Patients were recruited from two sites in Belgium and two sites in Italy.

(2) Patients meeting the inclusion and exclusion criteria as detailed in Supplementary Table 1 were eligible for inclusion in this trial.

Endpoints and Assessments

DLTs and Dose Modification

Patients experiencing DLT could continue treatment with BI 860585 at one dose level lower (not to be reduced below the starting dose; maximum two dose reductions allowed), providing sufficient recovery from drug-related toxicities occurred within 14 days, and as agreed by the investigator and sponsor.

Secondary Endpoints

(1) Duration of objective response: defined as the time from first objective response to the time of progression or death.

(2) Duration of clinical benefit: defined as time from first treatment administration until the earliest of disease progression or death, amongst patients with disease control.

Additional Pharmacokinetic Endpoints

Pharmacokinetic parameters were determined by non-compartmental analysis using Phoenix WinNonlin™ software (version Phoenix 6.3, Certara USA Inc., Princeton, NJ, USA). In the non-compartmental analysis, concentration data identified with no sample available (NOS), no valid result (NOR), and not analyzed (NOA) were not considered.

The following pharmacokinetic parameters were determined:

BI 860585:

(1) Pharmacokinetic parameters were assessed after single dose and at steady state for doses between 5 mg and 300 mg (Supplementary Table 2).

(2) Dose proportionality of the pharmacokinetic parameters maximum measured plasma concentration at steady state ($C_{max,ss}$), area under concentration–time curve between time 0 and ∞ ($AUC_{0-\infty}$), and steady-state area under concentration–time curve between 0 and 24h after dose ($AUC_{0-24,ss}$) of BI 860585 in cycle 1 of each treatment arm was assessed.

(3) The pharmacokinetic parameters of BI 860585 were assessed with and without food in pre-selected patients enrolled in Arm A on day 1 and day 2 of the first treatment cycle, for doses of 120 mg, 160 mg and 220 mg (intra-individual comparison).

Patients were assigned to treatment sequences ‘fasted-fed’ or ‘fed-fasted’ on two separate test days (fasted-fed group: overnight fast of ≥10 hours, followed by BI 860585 plus 240 mL water. Patients remained fasted until at least 2 hours after drug intake; limited water was permitted; fed-fasted

group: overnight fast of ≥10 hours and subsequent breakfast, then patients received BI 860585 with 240 mL water within 5 minutes of completion of breakfast). From day 3 to day 28 in cycle 1 and at all subsequent cycles, BI 860585 was taken after breakfast.

(4) Pharmacokinetic parameters $C_{max,ss}$ and $AUC_{0–24,ss}$ of BI 860585 as monotherapy and in combination with exemestane or with paclitaxel in cycle 1 of each treatment arm were assessed to investigate the potential influence of exemestane or paclitaxel on the pharmacokinetics of BI 860585 (inter-individual comparison),

Exemestane/Paclitaxel:

Pharmacokinetic parameters after once daily (exemestane) and once weekly (paclitaxel) dosing as single agents or in combination with BI 860585 at steady state were assessed to explore the potential effect of BI 860585 on exemestane and/or paclitaxel pharmacokinetics (intra-individual comparison; Supplementary Table 2).

Methods of Sample Collection

During the first treatment cycle, a total of 85–120 mL blood was taken from each patient for pharmacokinetic analysis (85 mL/120 mL for Arm A without/with food effect testing; 85 mL in Arms B and C). Approximately 4 mL of blood was collected for quantification of analyte plasma concentrations.

Analytical Determinations

BI 860585, paclitaxel and exemestane plasma concentrations were determined by a validated assay based on liquid chromatography coupled to tandem mass spectrometry. BI 860585 was analyzed at Boehringer Ingelheim (Germany) and paclitaxel and exemestane at Nuvisan GmbH (Germany).

Blood Sample Collection for Biomarker Analyses

Blood samples of approximately 5 mL were collected from all patients, in order to assess pharmacodynamic biomarkers related to the PI3K-AKT-mTOR signaling cascade, in platelet-rich plasma (PRP).

In Arm A (fed population), samples for PRP analysis were collected: during the screening period, pre- and post-BI 860585 administration on days 1 and 22 (1.0, 3.0 and 8.0 hours); pre- and post- BI 860585 administration on day 15 (3.0 hours); and pre-BI 860585 administration on days 2 and 23 of cycle 1.

In Arm A (food interaction cohort) samples for PRP analysis were collected: pre- and post-BI 860585 administration on days 1, 2 and 22 (1.0, 3.0 and 8.0 hours); and pre-BI 860585 administration on days 3 and 23 of cycle 1.

In Arm B, samples for PRP analysis were collected: during the screening period, pre-exemestane administration on day −7; pre- and post-drug administration on day 1 (1.0 and 3.0 hours); pre- and post-drug administration on day 15 (3.0 hours); pre- and post-drug administration on day 22 (1.0, 3.0 and 8.0 hours); and pre-drug administration of day 23 of cycle 1. Screening and day 15 samples were only collected from patients who had tumor biopsies.

In Arm C, samples for PRP analysis were collected: during the screening period, pre-paclitaxel infusion on day −7; pre- and post-drug administration on day 1 (1.0 and 3.0 hours); pre- and post-drug administration on day 15 (3.0 hours); pre- and post-drug administration on day 22 (1.0, 3.0 and 8.0 hours post-drug administration); and pre-drug administration of day 23 of cycle 1.

Table S1. Statistical Evaluation of the Effect of Food on Exposure to BI 860585 monotherapy.

	N (fed/fasted)	gMean ratio fed/fasted (%)	90% Confidence Interval		Intra-individual gCV (%)
			Lower limit	Upper limit	
120 mg					
C_{max}	7/5	85.38	76.47	95.34	7.4
$AUC_{0–24}$	6/5	92.67	82.63	103.92	7.6
$AUC_{0–\infty}$	5/5	91.25	77.56	107.37	10.7
160 mg					
C_{max}	3/3	96.18	82.56	112.04	2.8
$AUC_{0–24}$	3/3	91.94	87.71	96.37	0.9
$AUC_{0–\infty}$	3/3	82.02	53.90	124.80	7.7
220 mg					
C_{max}	6/7	83.90	73.21	96.16	10.5
$AUC_{0–24}$	5/7	94.76	83.68	107.32	7.9
$AUC_{0–\infty}$	5/7	82.90	67.54	101.74	11.5

Abbreviations: $AUC_{0–24}$, area under concentration–time curve between 0 and 24h after dose; $AUC_{0–\infty}$, area under concentration–time curve between time 0 and ∞; C_{max}, maximum plasma concentration; gCV, geometric coefficient of variation; gMean, geometric mean.

Table S2. Steady-State Pharmacokinetic Characteristics Obtained at Day 22 at the MTD in all Arms. All values are gMean (gCV [%]) unless otherwise indicated.

	**BI 860585 220 mg, Arm A (*n* = 5)**	**BI 860585 160 mg/ exemestane 25 mg, Arm B (*n* = 7)**	**BI 860585 160 mg/ paclitaxel 80 mg/m², Arm C (*n* = 3)**
$AUC_{0–24,ss}$ (mol·h/L)	838 (24.3)	988 (19.1)	730 (20.3)
$C_{max,ss}$ (mol/L)	50.5 (27.1)	61.4 (25.7)	35.4 (40.7)
$T_{max,ss}$ (h)†	3.0 (2.1–6.0)	2.0 (1.5–4.0)	6.0 (4.0–8.0)
$T_{1/2,ss}$ (h)	24.9 (22.2)	25.3 (25.9)	---
$R_{A,AUC}$	1.9 (20.6)	---	---

†Median (range). Abbreviations: $AUC_{0–24,ss}$, steady-state area under concentration–time curve between 0 and 24h after dose; $C_{max,ss}$, maximum steady-state plasma concentration; gMean, geometric mean; gCV, geometric coefficient of variation; h, hour; $R_{A,AUC}$, accumulation ratio based on AUC (ratio of steady-state AUC to AUC after single dose); $T_{max,ss}$, time to reach maximum plasma concentration at steady-state; $T_{1/2,ss}$, half-life at steady state.

Table S3. Proposed composition of the standard continental breakfast.

	**Amount (g)**	**Kcal**	**Fat (g)**	**Protein (g)**	**Carbs (g)**
1 egg	60.00	92.00	5.30	6.25	0.55
Saveloy/ham	25.00	107.50	9.75	5.25	0.17
Full-cream cheese (48% fat)	25.00	95.00	8.08	6.50	0
Butter	20.00	170.00	16.00	0.20	0
Two bread rolls	60.00	140.00	2.30	5.40	25.80
Jam	25.00	61.30	0.05	0.1	15.00
Honey	30.00	77.50	0	0	19.17
Banana	120.00	115.40	0.37	1.29	24.90
Cup (≈250 mL) of decaffeinated tea or coffee	0	0	0	0	0
Without banana but with egg		665.80	41.48	23.70	41.52
With banana but without egg		689.20	36.55	18.74	65.87
With honey instead of saveloy/ham (with egg, without banana)		635.80	6.55	18.74	84.87
SUM CTP		688.00	40.23	27.80	43.96

Table S4. Key Inclusion and Exclusion Criteria.

Key inclusion criteria	Key exclusion criteria
• Patients with histologically or cytologically confirmed diagnosis of progressive, advanced, measurable or evaluable, non-resectable, and/or metastatic solid tumors<br>• Patients who have received previous standard of care therapy for their disease and have progressed<br>• Age ≥18 years<br>• Life expectancy ≥3 months<br>• Written informed consent in accordance with International Conference on Harmonization/Good Clinical Practice and local legislation<br>• ECOG PS ≤2<br><br><u>Additional inclusion criteria for the combination arms:</u><br>• Patients must have confirmed progressive disease within the last 6 months (in case of measurable disease, progression should be confirmed according to RECIST criteria 1.1)<br>• Patients for whom treatment with either exemestane or paclitaxel would be considered appropriate by the investigator	• Serious concomitant non-oncological disease considered by the investigator to be incompatible with the protocol<br>• Patients with untreated or symptomatic brain metastases unless they are stable (defined as no change on CT scan or MRI for at least two months and no change in steroid dose for at least four weeks, unless change due to intercurrent infection or other acute event) or have normal brain MRI scan at screening and be at least 4 weeks post radiation or surgery for brain metastasis<br>• Second malignancies requiring active therapy except for adequately resected cervix carcinoma in situ, and resected non-melanomatous skin cancers (including basal cell carcinoma and squamous cell arcinoma)<br>• Clinical congestive heart failure grade III–IV<br>• Myocardial infarction within the last 6 months prior to inclusion, or symptomatic coronary artery disease<br>• Absolute neutrophil count <1,500/mm^3<br>• Platelet count <100,000/mm^3<br>• Total bilirubin >1.5 x upper limit of normal value (including known Gilbert's syndrome)<br>• Aspartate amino transferase (AST) and/or alanine amino transferase (ALT) >3 x upper limit of normal value (if related to liver metastases greater than 5 x upper limit of normal value)<br>• Serum creatinine >1.5 x upper limit of normal value<br>• Pregnancy or breastfeeding<br>• Women or men who are sexually active and unwilling to use a medically acceptable method of contraception<br>• Patients unable to comply with the protocol<br>• Known or suspected active drug or alcohol abuse<br>• Patients with known HIV/hepatitis/active infectious disease considered by the investigator to be incompatible with the protocol<br>• Patients unable to take oral medication (BI 860585 may not be crushed or administered via a gastrostomy tube)<br>• Chronic diarrhea or other gastrointestinal disorders that may interfere with the absorption of the study medication<br>• Treatment with anti-cancer-therapies: cytotoxic or standard chemotherapy, immunotherapy, radiotherapy, biological therapies, molecular targeted or other investigational drugs, within 4 weeks of the first treatment with the study medication (or within 1 week for non-cytotoxic drugs)<br>• Major surgery within the last 28 days prior to start of trial medication, or unresolved effects of any previous surgery in the opinion of the investigator<br>• Continuation of CTCAE grade ≥2 therapy-related toxicities from prior anti-cancer therapies or prior surgery, at the time of the first administration of the study medication (except alopecia)<br>• Hypersensitivity to combination drugs or excipients<br>• Patients with a history of uncontrolled diabetes mellitus

Table S5. Blood Sampling Schedule for Pharmacokinetic Analyses During.

**Arm**	**Dose Status**	**Days into the Trial**												
		**−7**	**−6**	**−5**	**−1**	**1**	**2**	**3**	**8**	**15**	**22**	**23**	**24**	**28**
**A**	Pre-BI 860585					X	X		X	X	X	X		X
	Post-BI 860585†					X					X			
	Pre-BI 860585 (food interaction cohort only)							X						
	Post-BI 860585 (food interaction cohort only)†						X							
B	Pre-exemestane	X			X									
	Post-exemestane†				X									
	Pre-exemestane + BI 860585					X					X	X		X
	Post-exemestane + BI 860585†										X			
C	Pre-paclitaxel	X												
	During paclitaxel‡	X												
	Post-paclitaxel§	X	X	X										
	Pre-paclitaxel + BI 860585					X					X			X
	During paclitaxel + BI 860585‡										X			
	Post-paclitaxel + BI 860585†										X			
	Pre-BI 860585											X	X	

Cycle 1: †Sampled at 0.5, 1.0, 2.0, 3.0, 4.0, 6.0 and 8.0 hours. ‡Sampled at start of, and directly before the end of, paclitaxel infusion. §Sampled at 2.0, 4.0 and 6.0 hours on day −7, and at 0 hours on days −6 and −5.

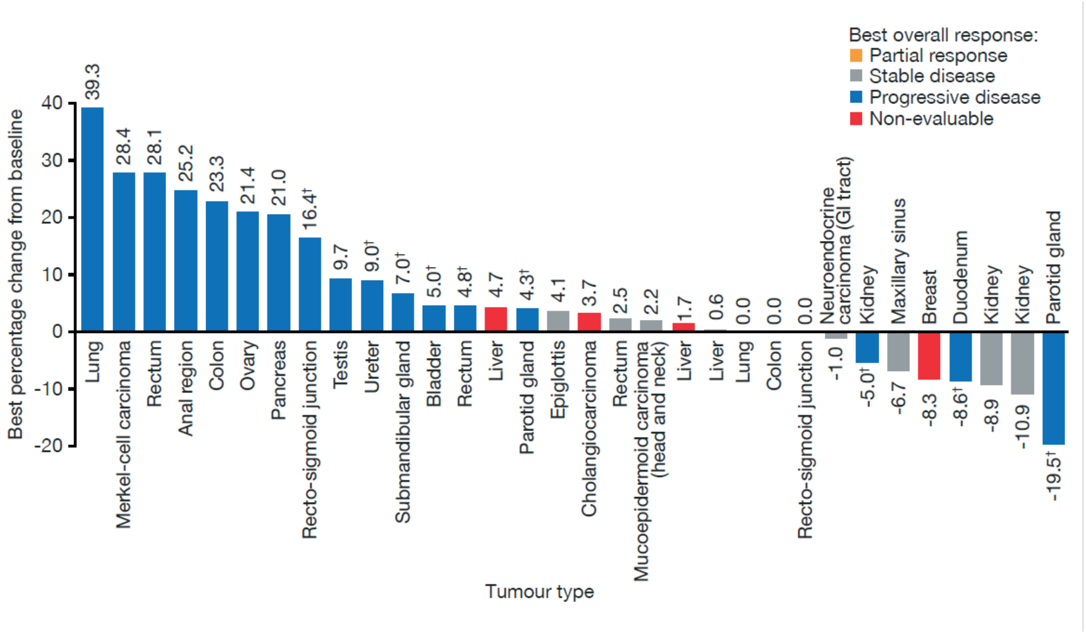

Figure S1. A. Best Percentage Change From Baseline in Target Lesions (Arm A: Monotherapy)*, *Percentage change data not available for 9 patients (non-evaluable, n=4; best overall response PD, n=5 [non-target lesions, n=3; non-evaluable target lesions, n=2]). Abbreviations: GI, gastrointestinal. †PD due to the development of a new lesion, or the progression of a non-target lesion.

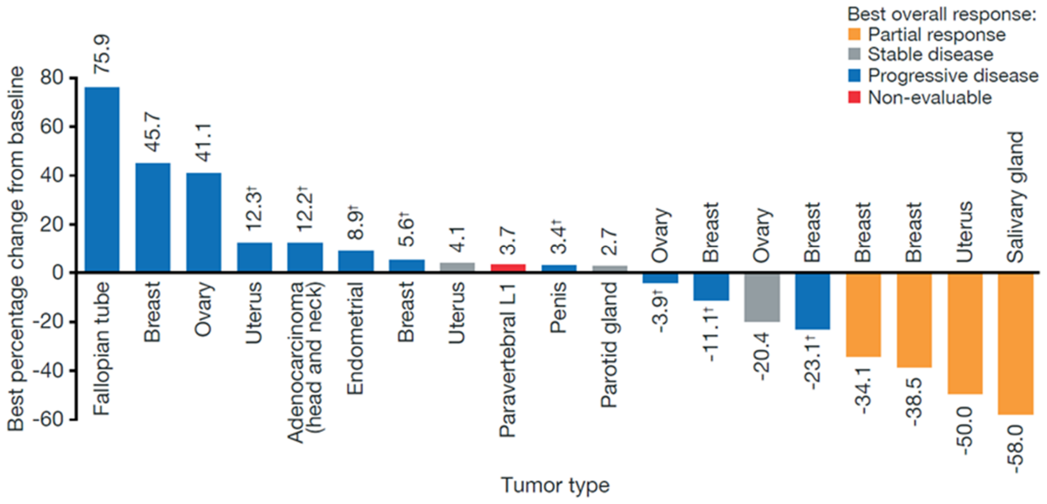

Figure S1. B. Best Percentage Change from Baseline in Target Lesions (Arm B: Combination with Exemestane)*, *Data not available for 6 patients (non-evaluable, n=4; best overall response PD, n=1 [non-target lesions]; best overall response non-CR/non-PD, n=1 [non-target lesions]). †PD due to the development of new lesion, or the progression of non-target lesions

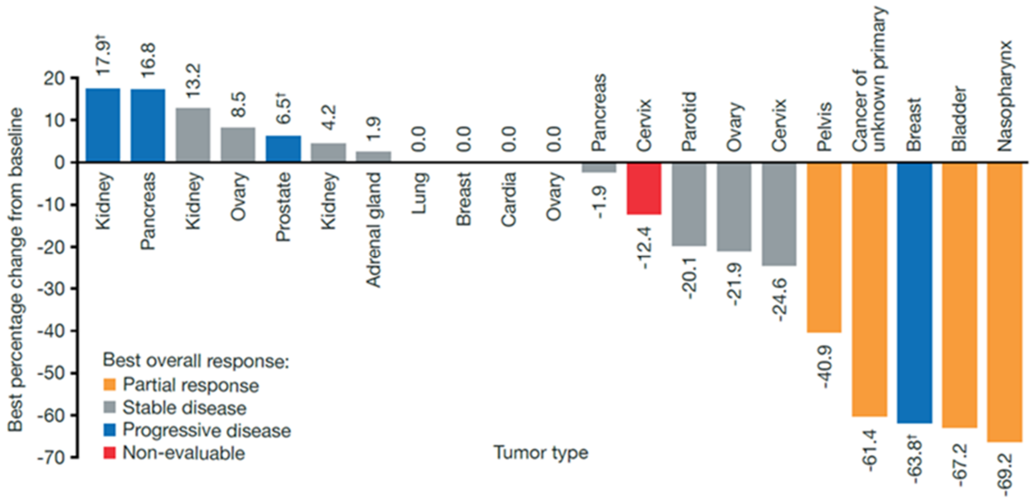

Figure S1. C. Best Percentage Change from Baseline in Target Lesions (Arm C: Combination with Paclitaxel)*, *Percentage change data are not available for three patients (non-evaluable n=1; best overall response of non-CR/non-PD [only non-target lesions] n=1; best overall response of CR [only non-target lesions] n=1). †PD due to the development of new lesion, or the progression of non-target lesions.

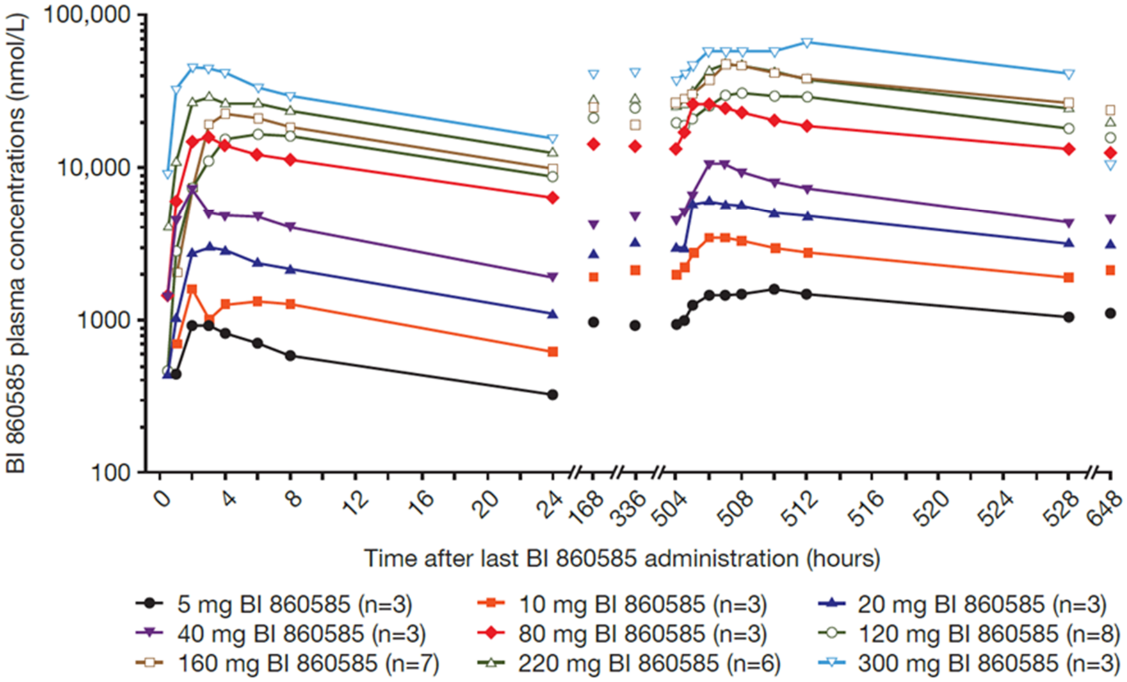

Figure S2. Plasma concentration-time profiles after once-daily administration of 5 to 300 mg BI 860585 (semi-logarithmic scale).

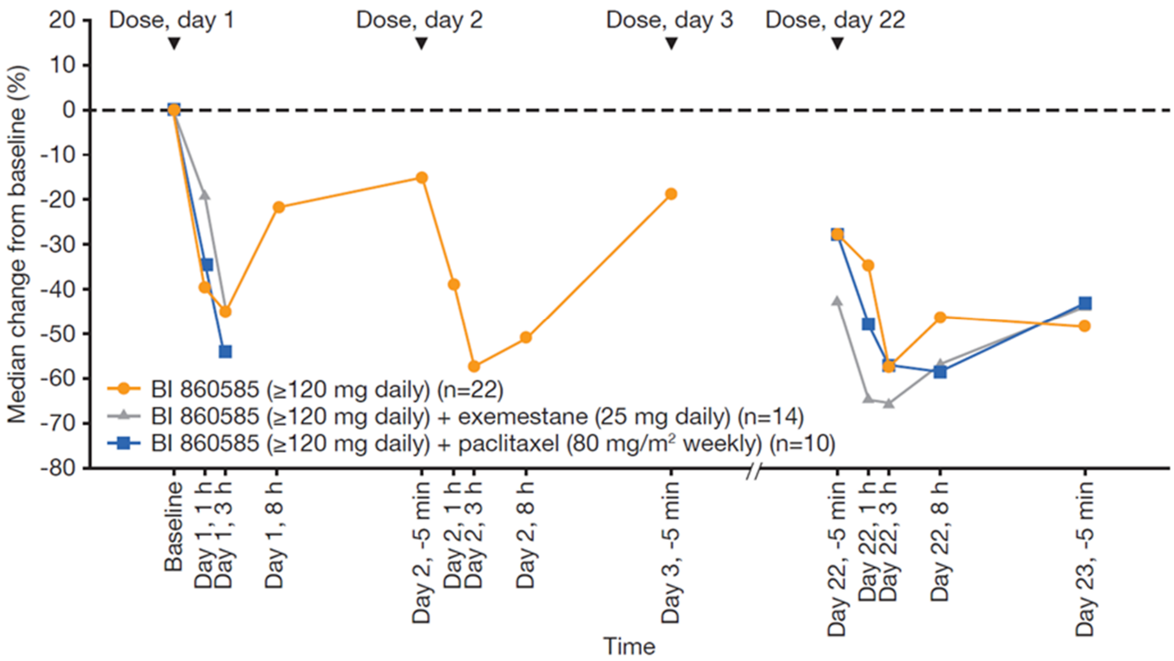

Figure S3. Median Change from Baseline in pAKT/AKT Ratio.

© 2020 by the authors. Submitted for possible open access publication under the terms and conditions of the Creative Commons Attribution (CC BY) license (http://creativecommons.org/licenses/by/4.0/).
